# Supplementary figures and images for: Genome-wide profiling of RNA 2’-O-methylation in neurons and identification of orphan snoRNA targets
Source: bioRxiv. 2025 Dec 17:2025.12.17.694928. Preprint. [Version 1] doi: 10.64898/2025.12.17.694928 (PMC12724518; doi:10.64898/2025.12.17.694928)

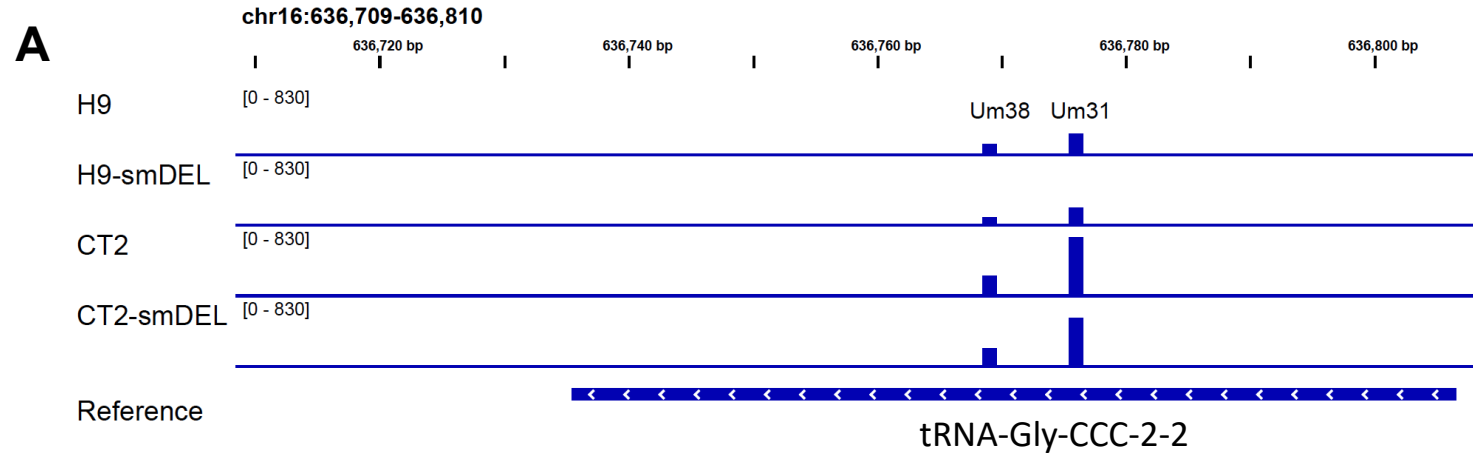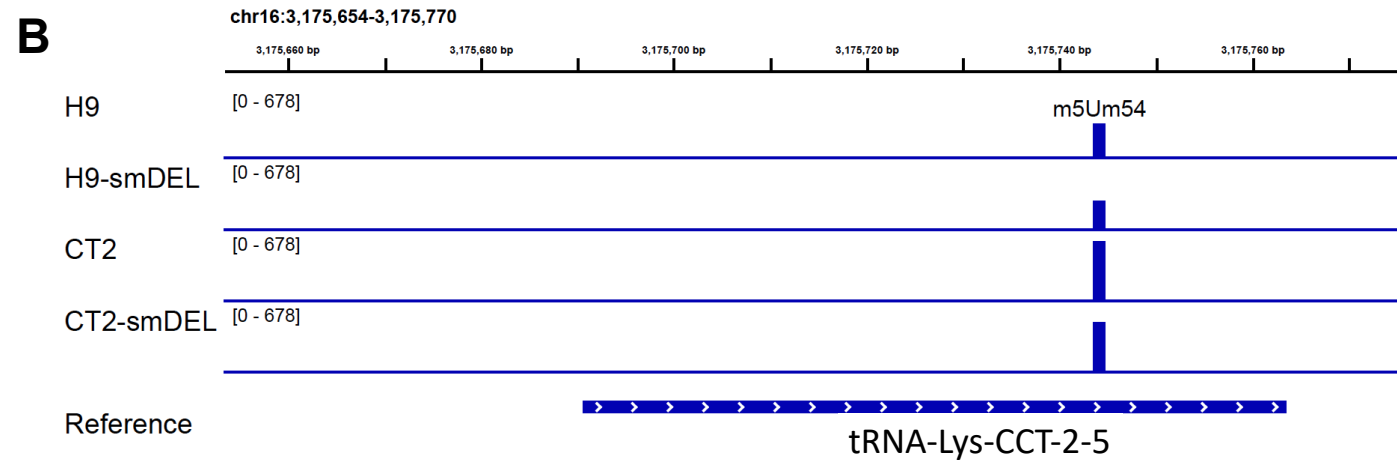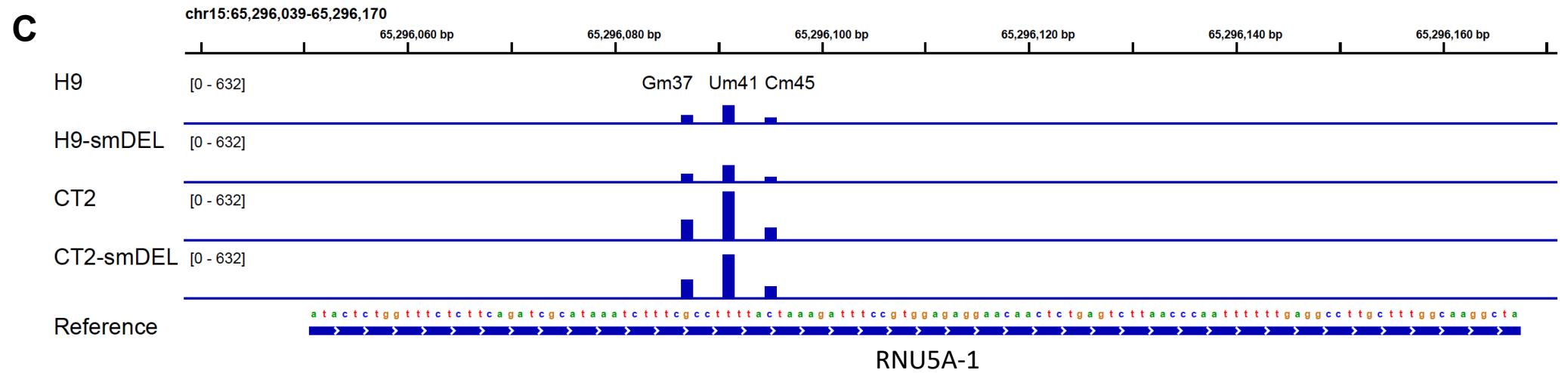

D

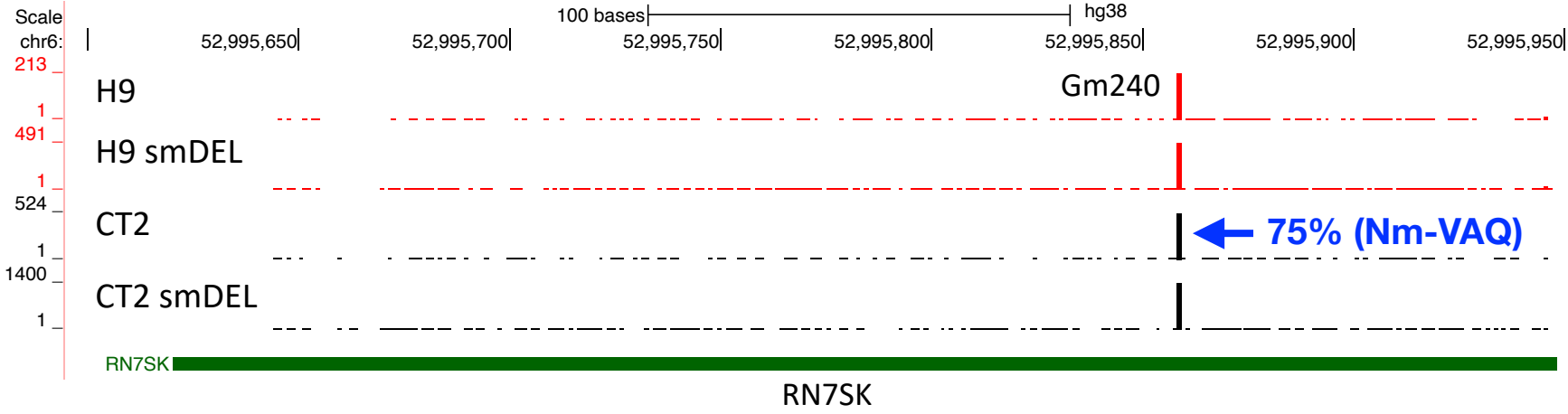

Supplement: Supplement 1 — Figure S1. RibOxi-seq2 peaks in tRNA, snRNA, RN7SK from induced neurons. Modified nucleotides annotated in reference are highlighted. The RN7SK Nm site was quantified at 75% using Nm-VAQ. [file media-1.pdf]

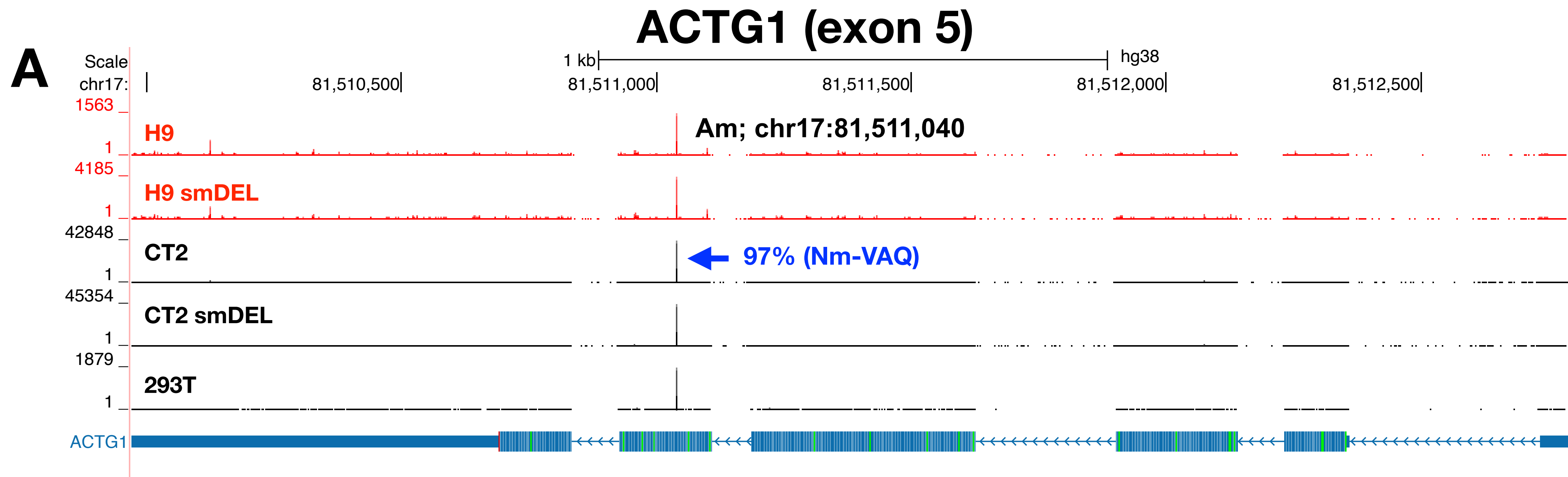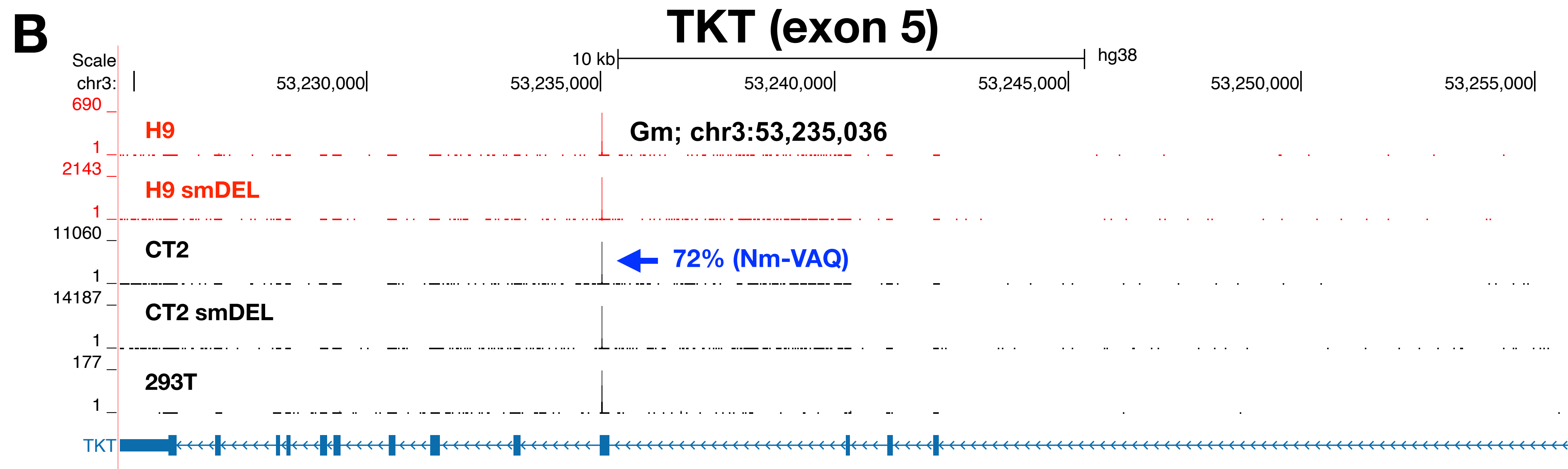

# NUDT21 (exon 1)

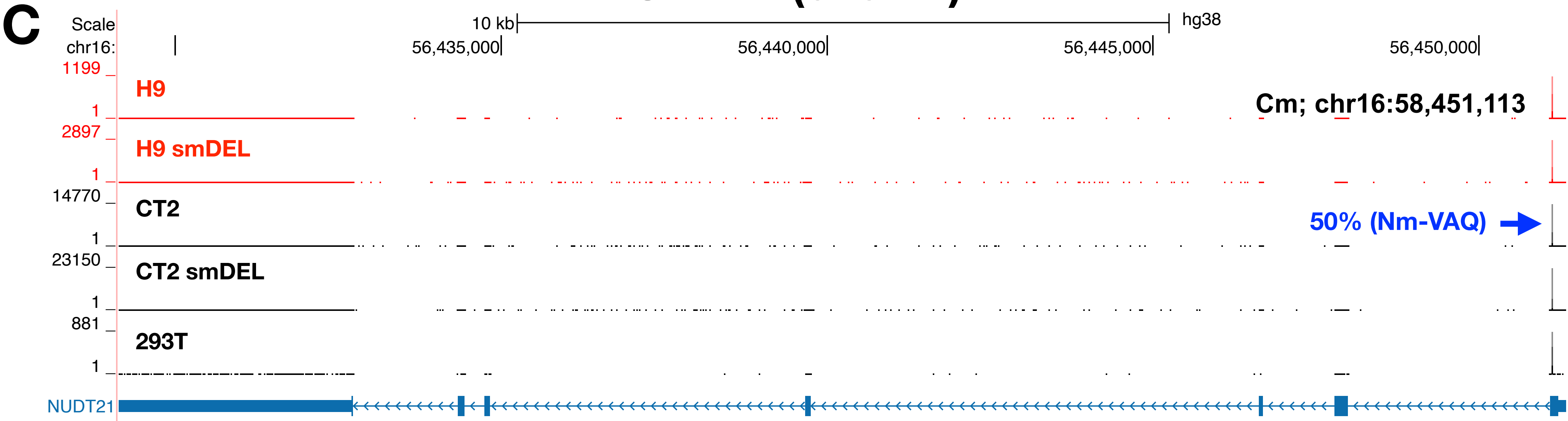

# NEFM (exon 1)

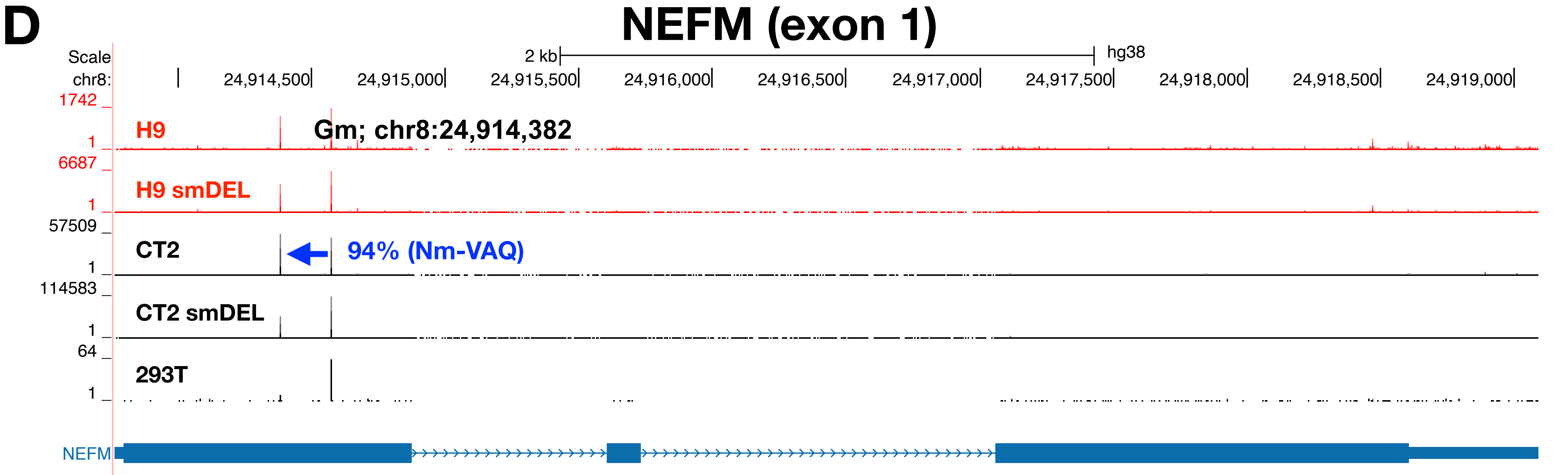

E

# AUP1 (exon 3)

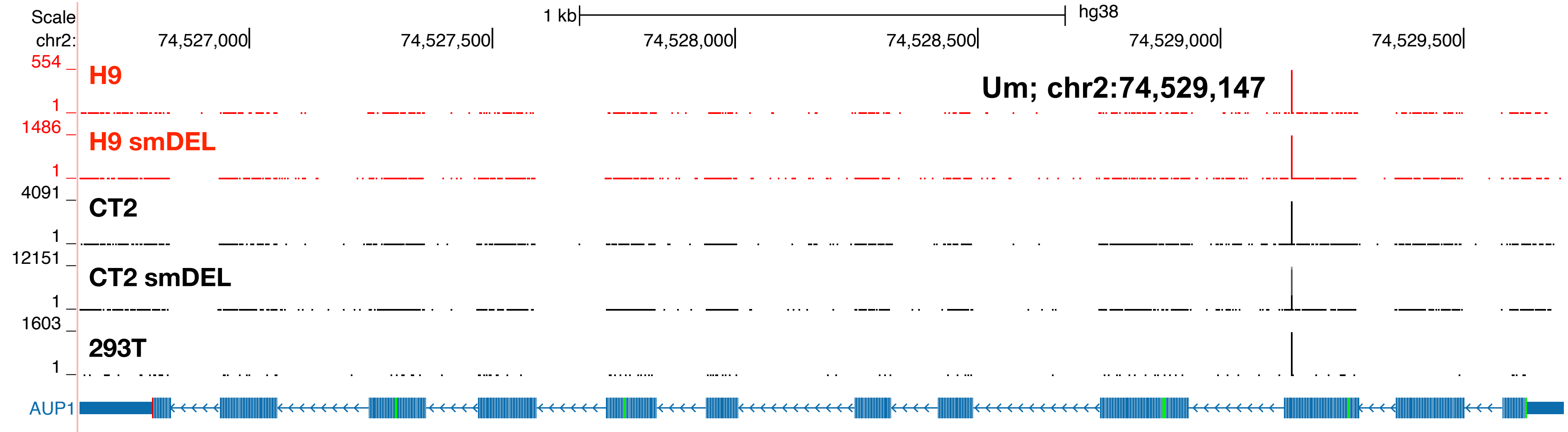

F

# CCT3 (exon 7)

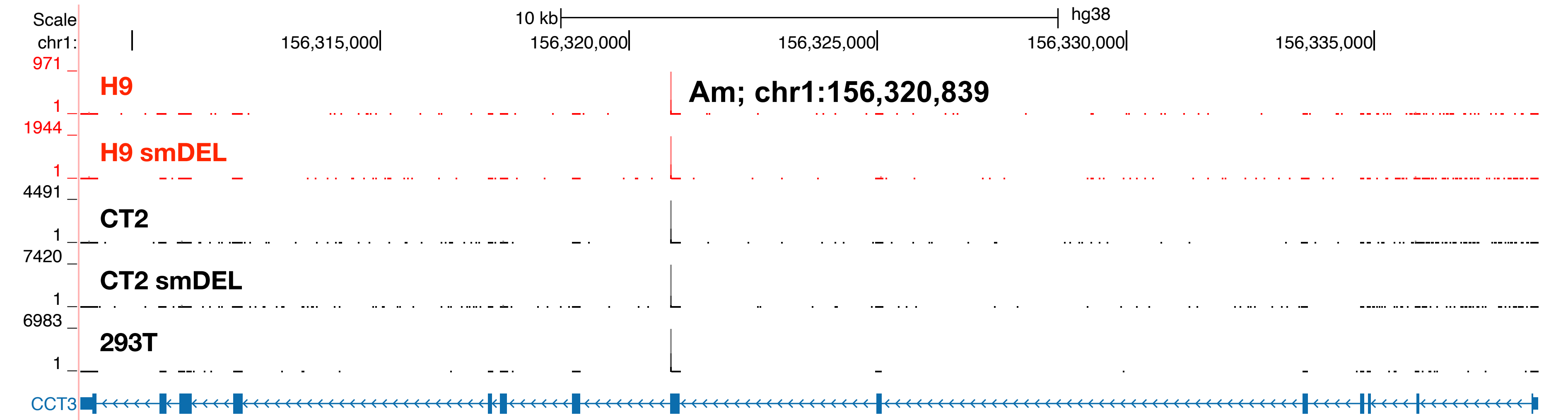

G

## ENY2 (3'-UTR)

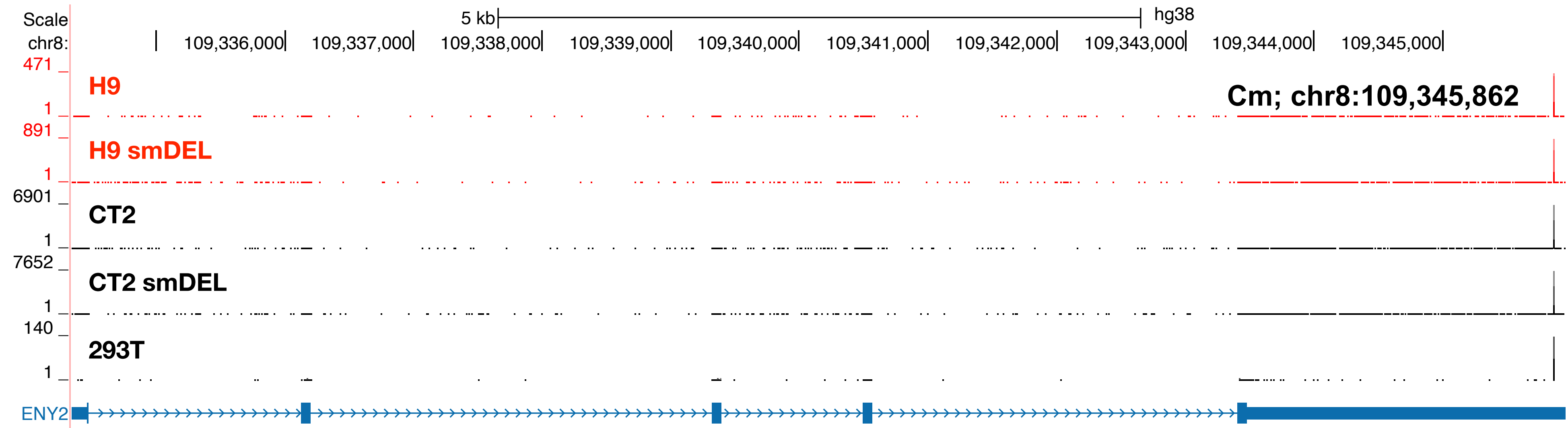

H

## HIRA (exon 19)

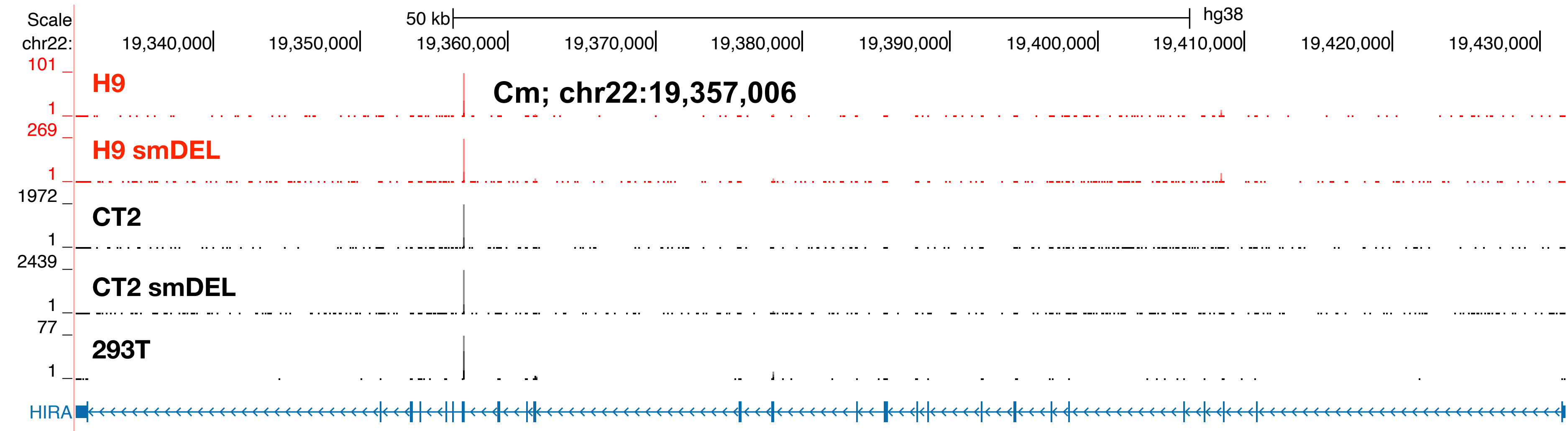

# LSM1 (3'-UTR)

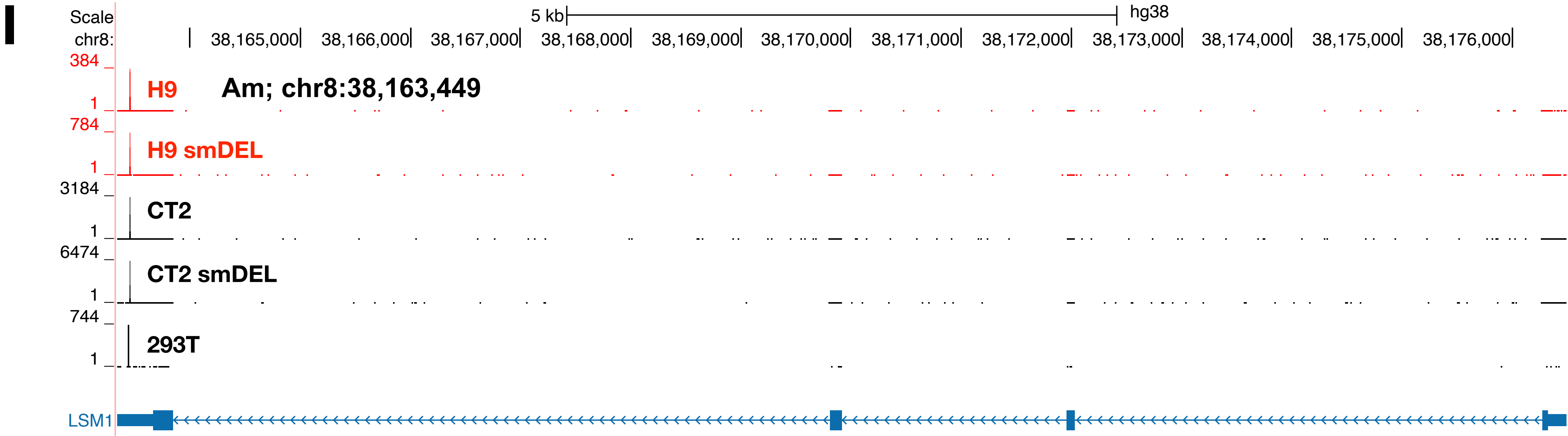

# NRBP1 (exon 7)

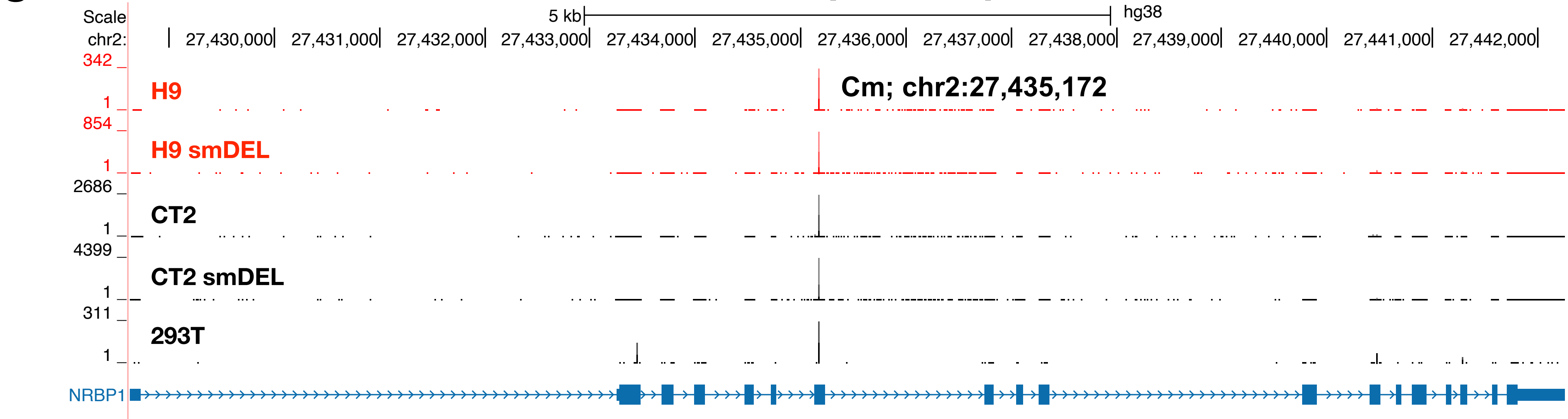

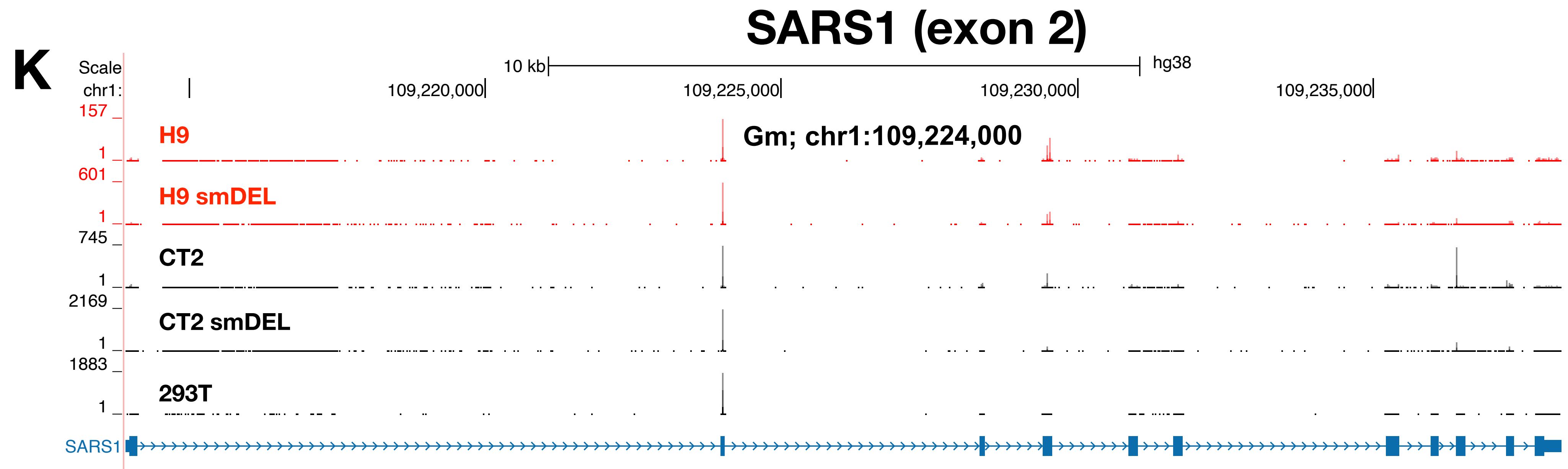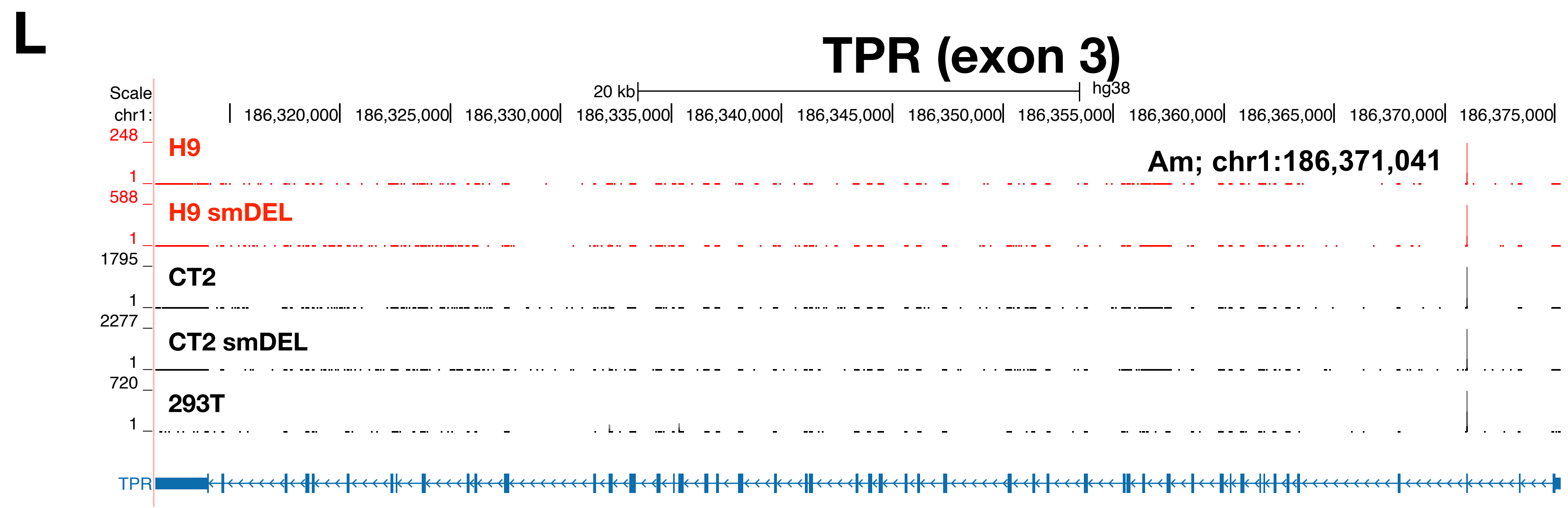

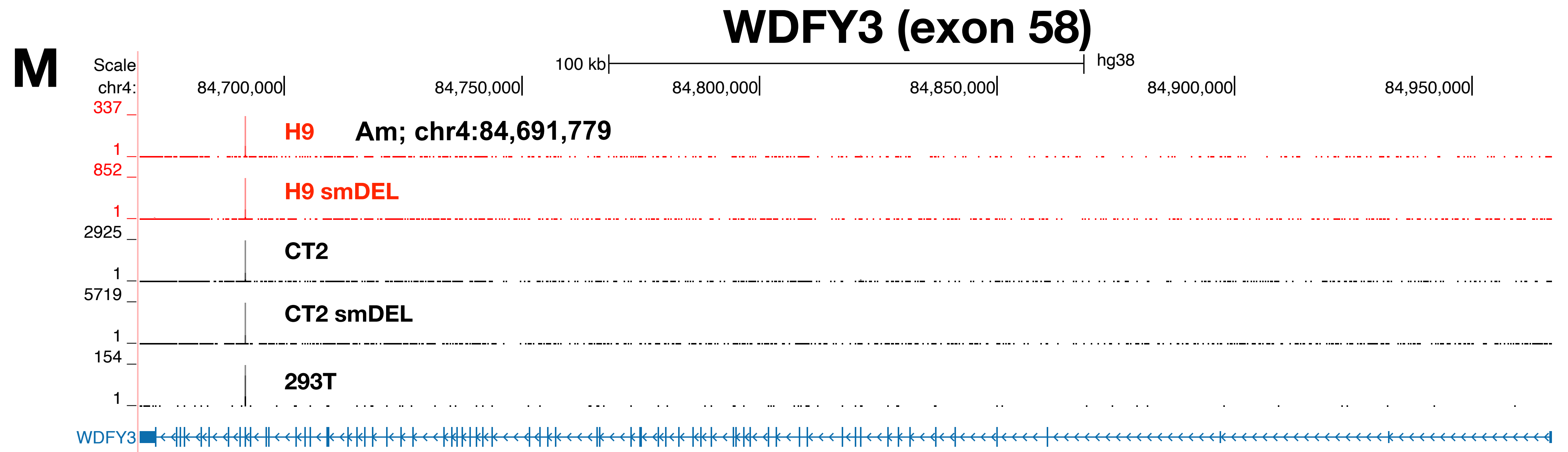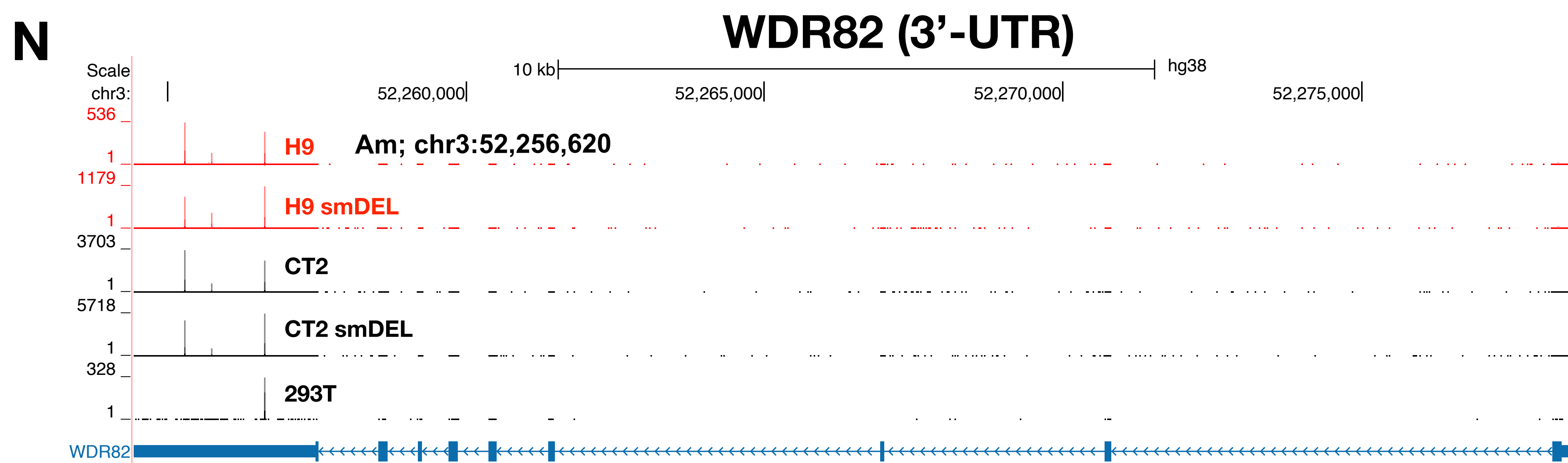

Supplement: Supplement 2 — Figure S2. Some additional representative examples of Nm sites in mRNAs, illustrating similar modification profiles between dirrerent cells. Percentages adjacent to some peaks denote modification levels quantified by Nm-VAQ. [file media-2.pdf]

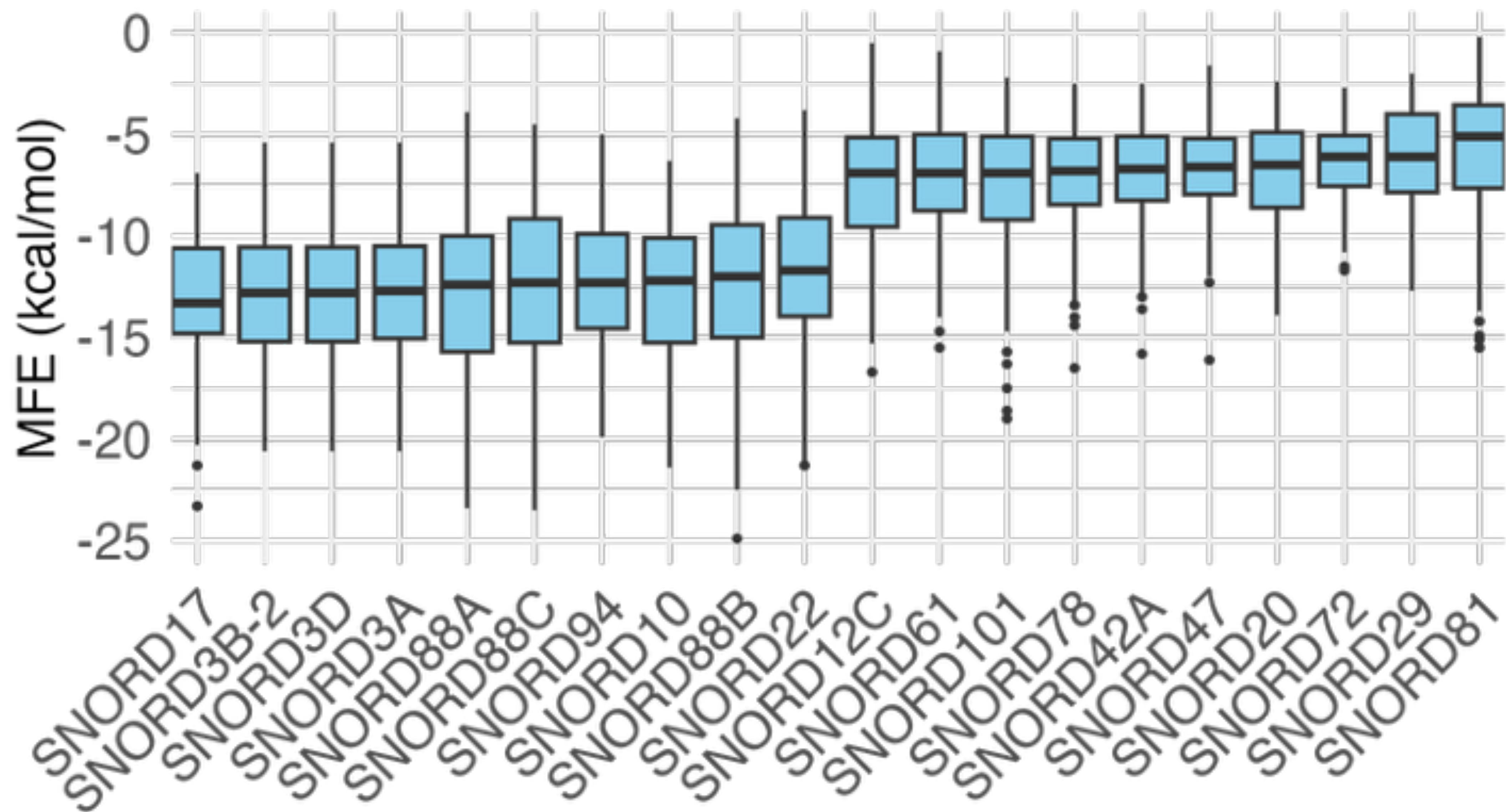

Supplement: Supplement 3 — Figure S3. Box plot comparison of minimum folding energy (MFE) across snoRNAs and RNA regions encompassing each Nm site (±7 nt). For each snoRNA species, the potential MFE values are shown; boxes denote the 25th–75th percentiles with a line at the median, and whiskers indicate 1.5× the interquartile range. [file media-3.pdf]
