# Supplementary material for: Genome-wide profiling of RNA 2’-O-methylation in neurons and identification of orphan snoRNA targets
Source: bioRxiv. 2025 Dec 17:2025.12.17.694928. Preprint. [Version 1] doi: 10.64898/2025.12.17.694928 (PMC12724518; doi:10.64898/2025.12.17.694928)
Supplement: Supplement 4 — Figure S4. Representative UCSC genome browser images illustrating bigWig tracks from CT2 and H9 induced neurons as well as small-deletion induced neurons and, in almost all cases, 293T cells. Nm positions are denoted by RibOxi-seq2 peaks, while U3 snoRNA CLASH fragment overlaps from HEK293T cells are shown at the top of each panel. [file media-4.pdf]

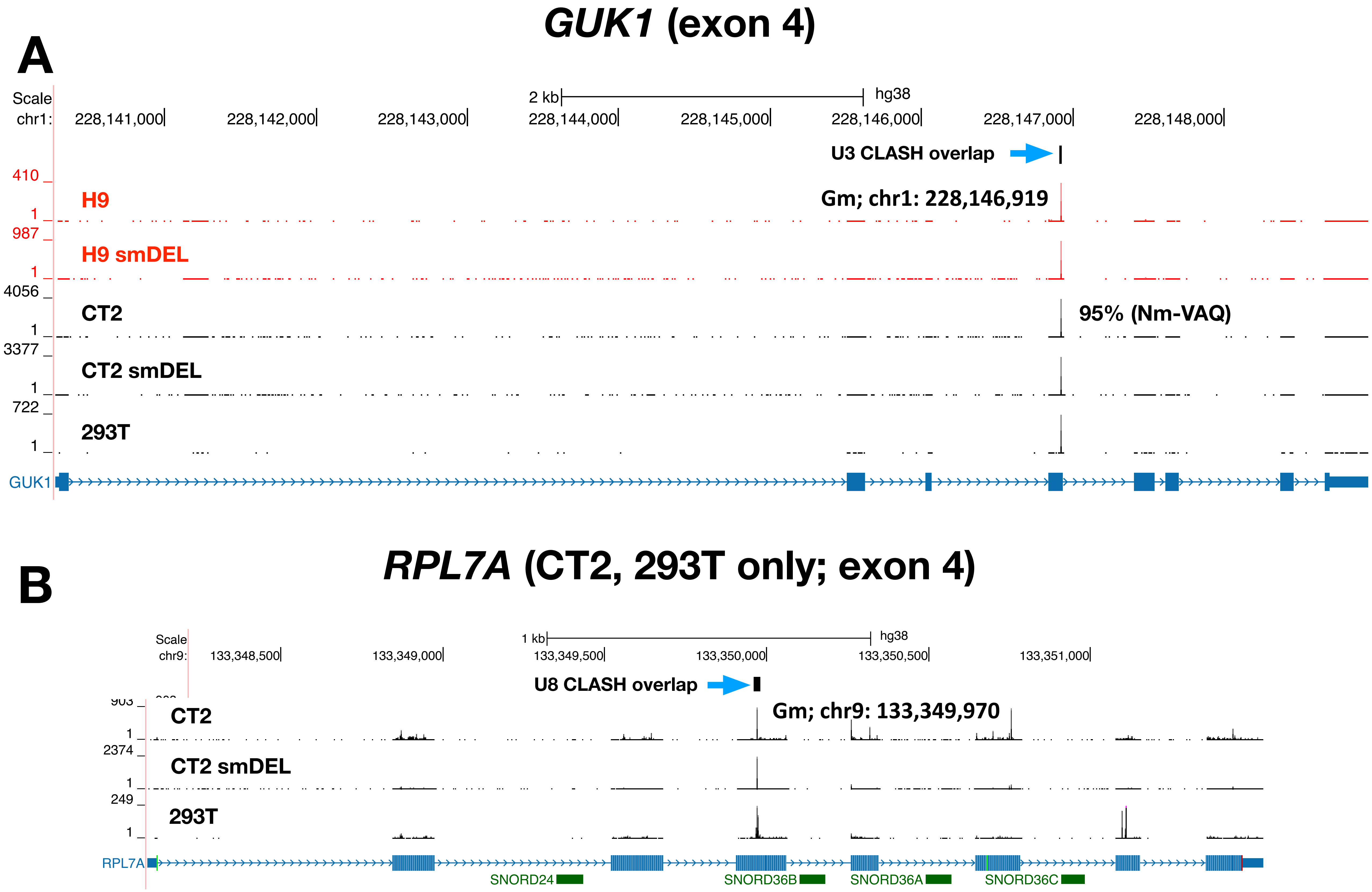
GUK1 (exon 4)
A
Scale
chr1:
2 kb
hg38
228,141,000
228,142,000
228,143,000
228,144,000
228,145,000
228,146,000
228,147,000
228,148,000
U3 CLASH overlap
410
H9
Gm; chr1: 228,146,919
1
987
H9 smDEL
1
4056
CT2
95% (Nm-VAQ)
1
3377
CT2 smDEL
1
722
293T
1
GUK1
RPL7A (CT2, 293T only; exon 4)
B
Scale
chr9:
1 kb
hg38
133,348,500
133,349,000
133,349,500
133,350,000
133,350,500
133,351,000
U8 CLASH overlap
CT2
Gm; chr9: 133,349,970
1
2374
CT2 smDEL
1
249
293T
1
RPL7A
SNORD24
SNORD36B
SNORD36A
SNORD36C

C

AZIN1 (exon 6)

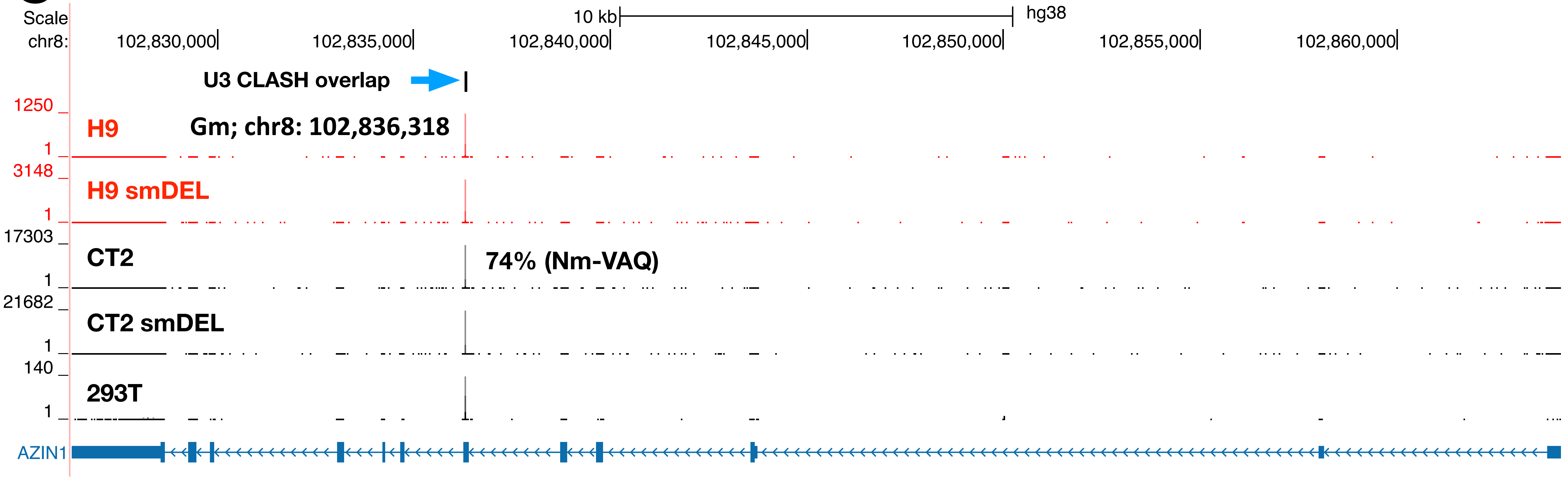

D

KMT2A (H9 only; exon 27)

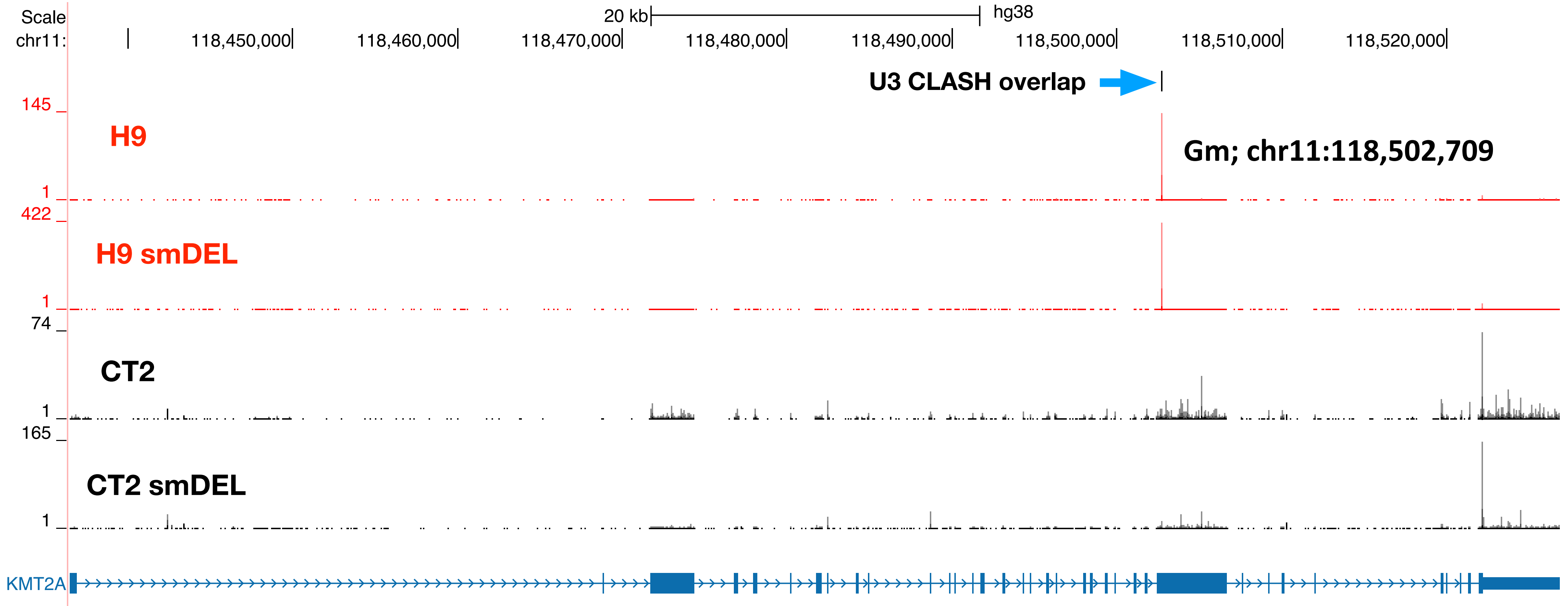

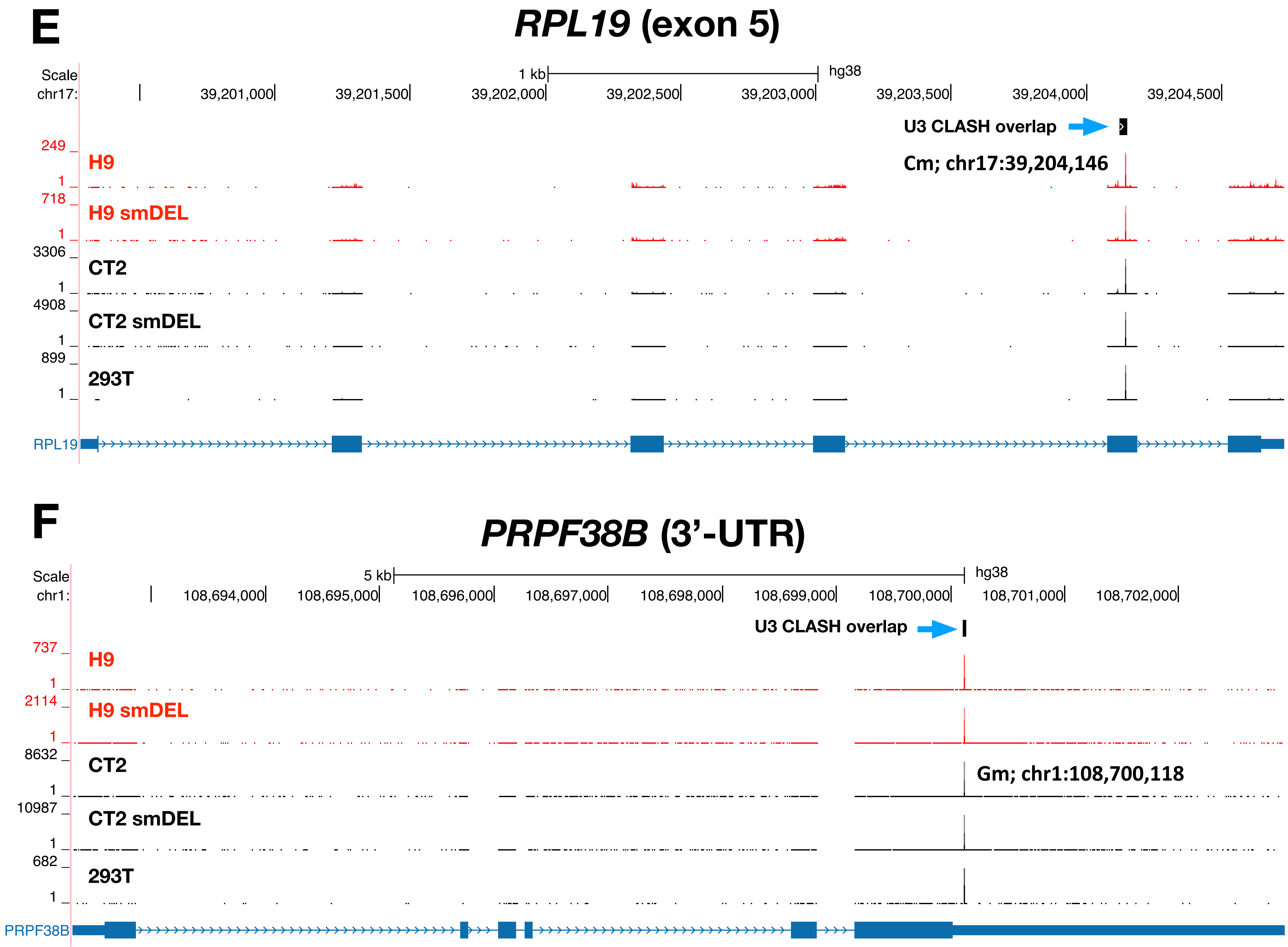
E
RPL19 (exon 5)
Scale
chr17:
1 kb
hg38
39,201,000
39,201,500
39,202,000
39,202,500
39,203,000
39,203,500
39,204,000
39,204,500
U3 CLASH overlap
Cm; chr17:39,204,146
249
1
H9
718
1
H9 smDEL
3306
1
CT2
4908
1
CT2 smDEL
899
1
293T
RPL19
F
PRPF38B (3'-UTR)
Scale
chr1:
5 kb
hg38
108,694,000
108,695,000
108,696,000
108,697,000
108,698,000
108,699,000
108,700,000
108,701,000
108,702,000
U3 CLASH overlap
737
1
H9
2114
1
H9 smDEL
8632
1
CT2
Gm; chr1:108,700,118
10987
1
CT2 smDEL
682
1
293T
PRPF38B

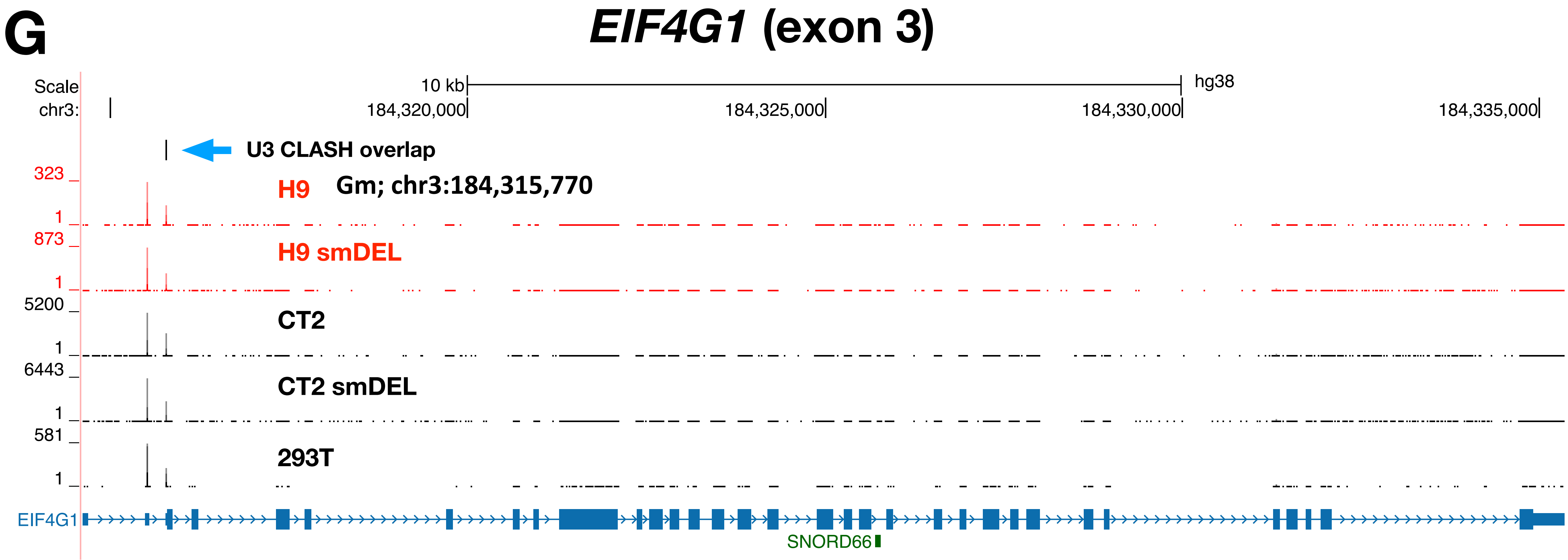
G
EIF4G1 (exon 3)
Scale
chr3:
10 kb
hg38
184,320,000
184,325,000
184,330,000
184,335,000
U3 CLASH overlap
323
1
H9
Gm; chr3:184,315,770
873
1
H9 smDEL
5200
1
CT2
6443
1
CT2 smDEL
581
1
293T
EIF4G1
SNORD66

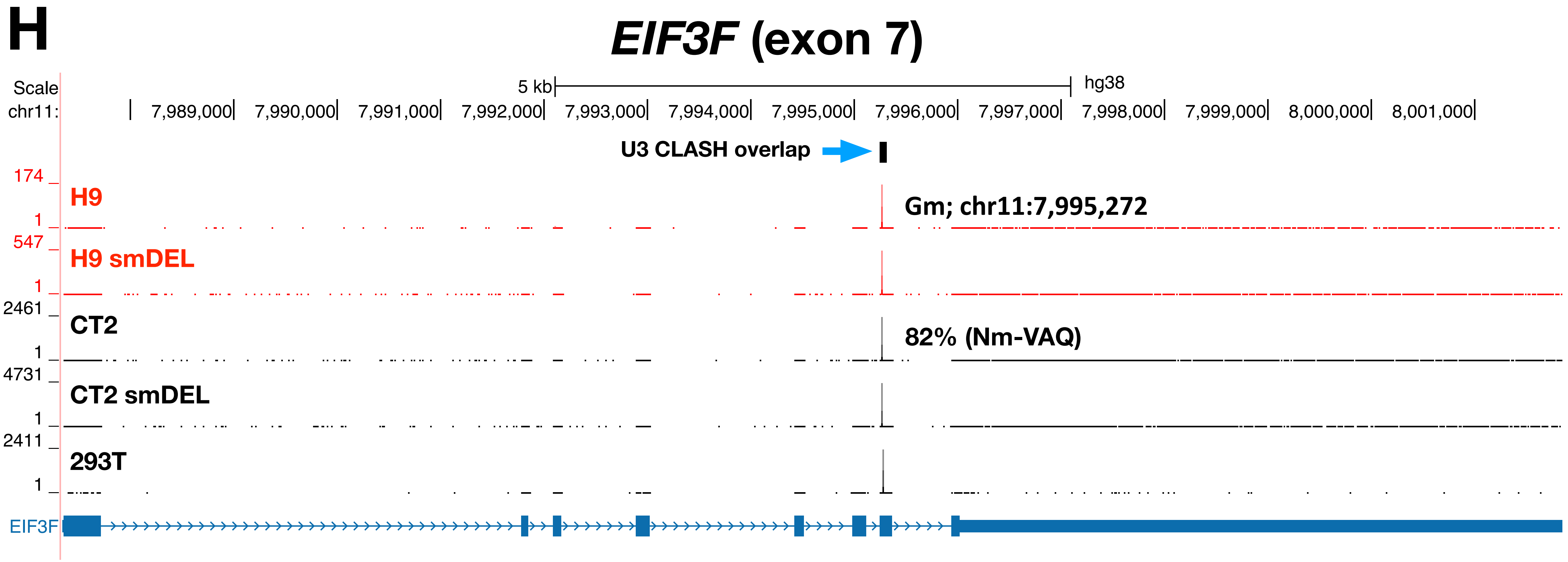
H
EIF3F (exon 7)
Scale
chr11:
5 kb
hg38
7,989,000
7,990,000
7,991,000
7,992,000
7,993,000
7,994,000
7,995,000
7,996,000
7,997,000
7,998,000
7,999,000
8,000,000
8,001,000
U3 CLASH overlap
174
1
H9
Gm; chr11:7,995,272
547
1
H9 smDEL
2461
1
CT2
82% (Nm-VAQ)
4731
1
CT2 smDEL
2411
1
293T
EIF3F

I

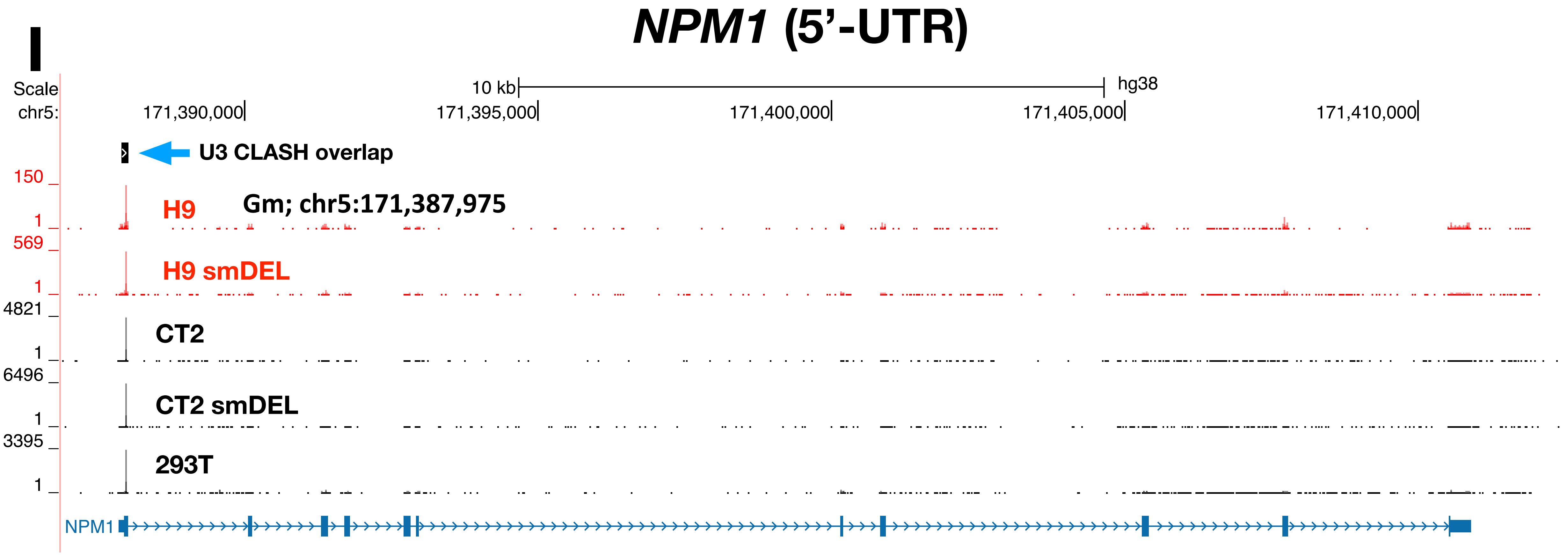
NPM1 (5'-UTR)
Scale
chr5:
10 kb
hg38
171,390,000
171,395,000
171,400,000
171,405,000
171,410,000
U3 CLASH overlap
150
1
H9
Gm; chr5:171,387,975
569
1
H9 smDEL
4821
1
CT2
6496
1
CT2 smDEL
3395
1
293T
NPM1

J

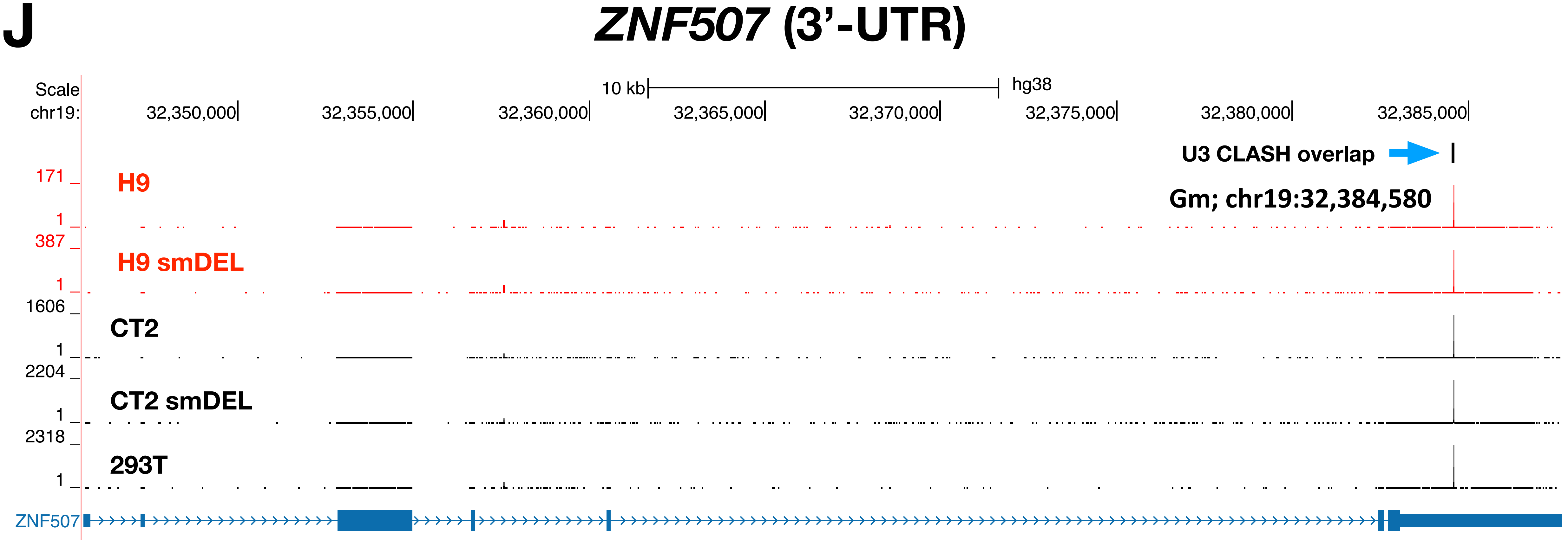
ZNF507 (3'-UTR)
Scale
chr19:
10 kb
hg38
32,350,000
32,355,000
32,360,000
32,365,000
32,370,000
32,375,000
32,380,000
32,385,000
U3 CLASH overlap
171
1
H9
Gm; chr19:32,384,580
387
1
H9 smDEL
1606
1
CT2
2204
1
CT2 smDEL
2318
1
293T
ZNF507

K

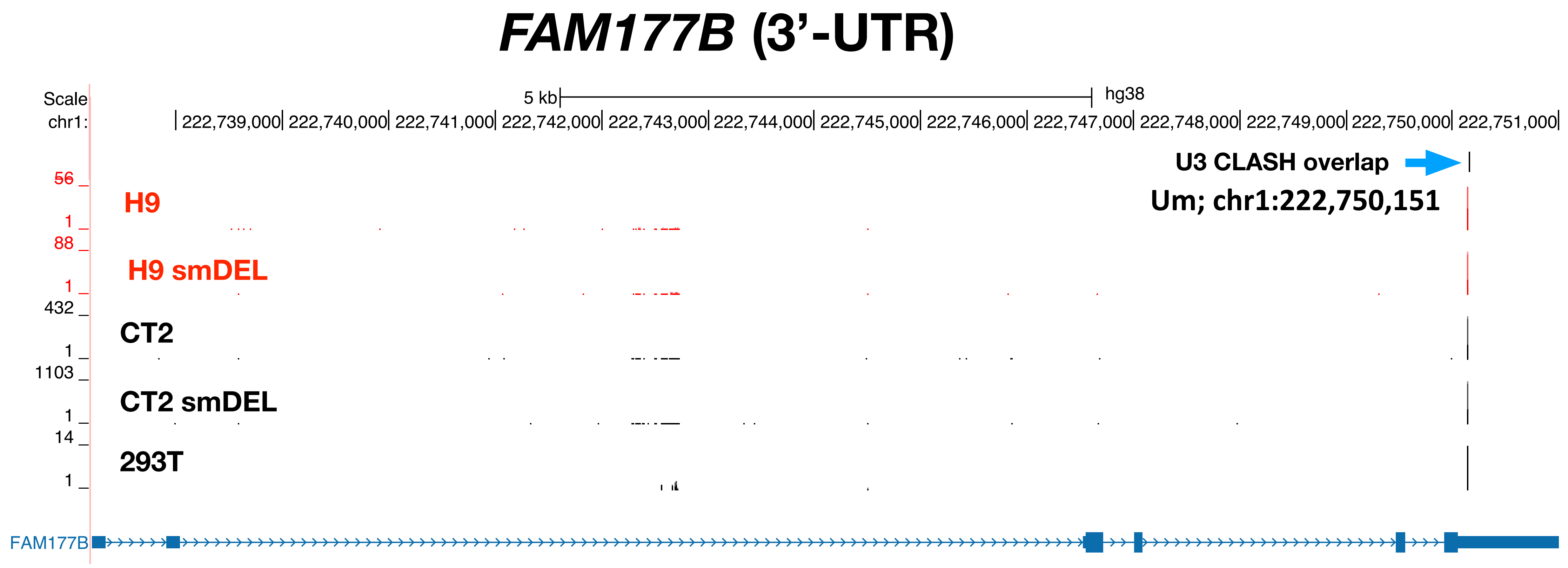
FAM177B (3'-UTR)
Scale
chr1:
5 kb
hg38
222,739,000 222,740,000 222,741,000 222,742,000 222,743,000 222,744,000 222,745,000 222,746,000 222,747,000 222,748,000 222,749,000 222,750,000 222,751,000
U3 CLASH overlap
Um; chr1:222,750,151
56
H9
1
88
H9 smDEL
1
432
CT2
1
1103
CT2 smDEL
1
14
293T
1
FAM177B

L

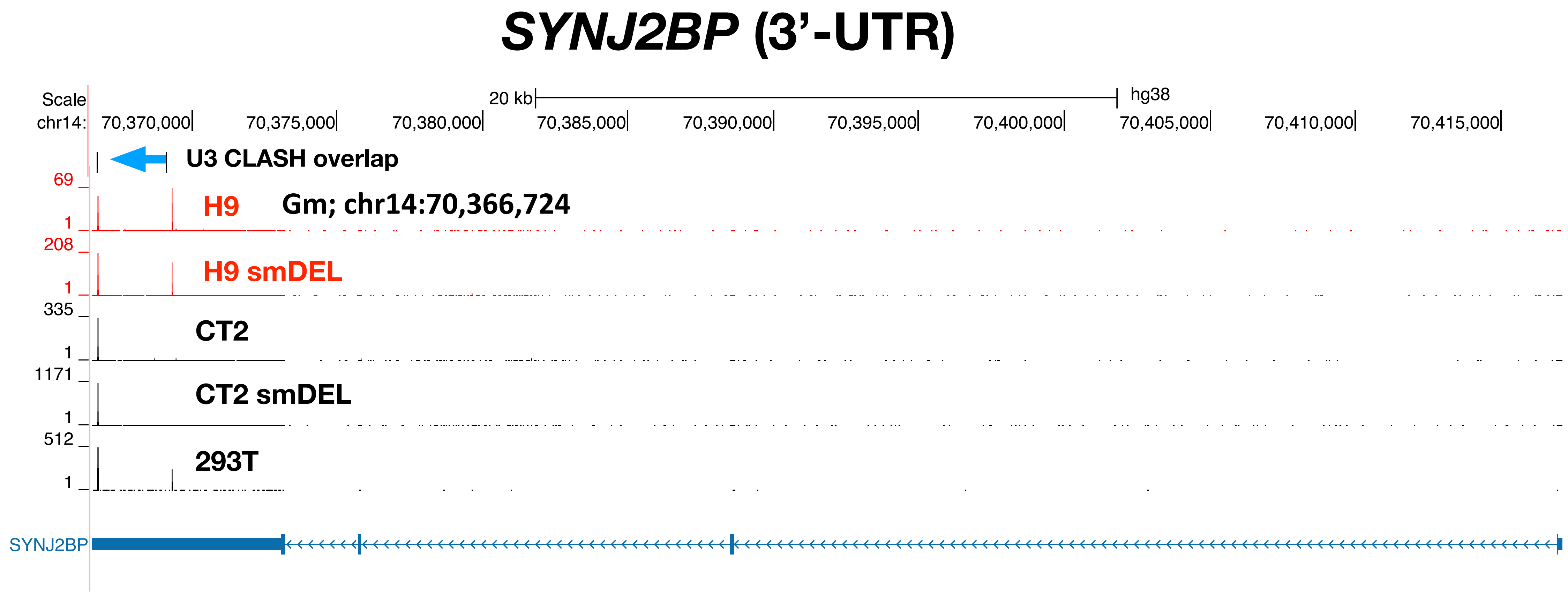
SYNJ2BP (3'-UTR)
Scale
chr14:
20 kb
hg38
70,370,000 70,375,000 70,380,000 70,385,000 70,390,000 70,395,000 70,400,000 70,405,000 70,410,000 70,415,000
U3 CLASH overlap
69
H9
Gm; chr14:70,366,724
1
208
H9 smDEL
1
335
CT2
1
1171
CT2 smDEL
1
512
293T
1
SYNJ2BP

M

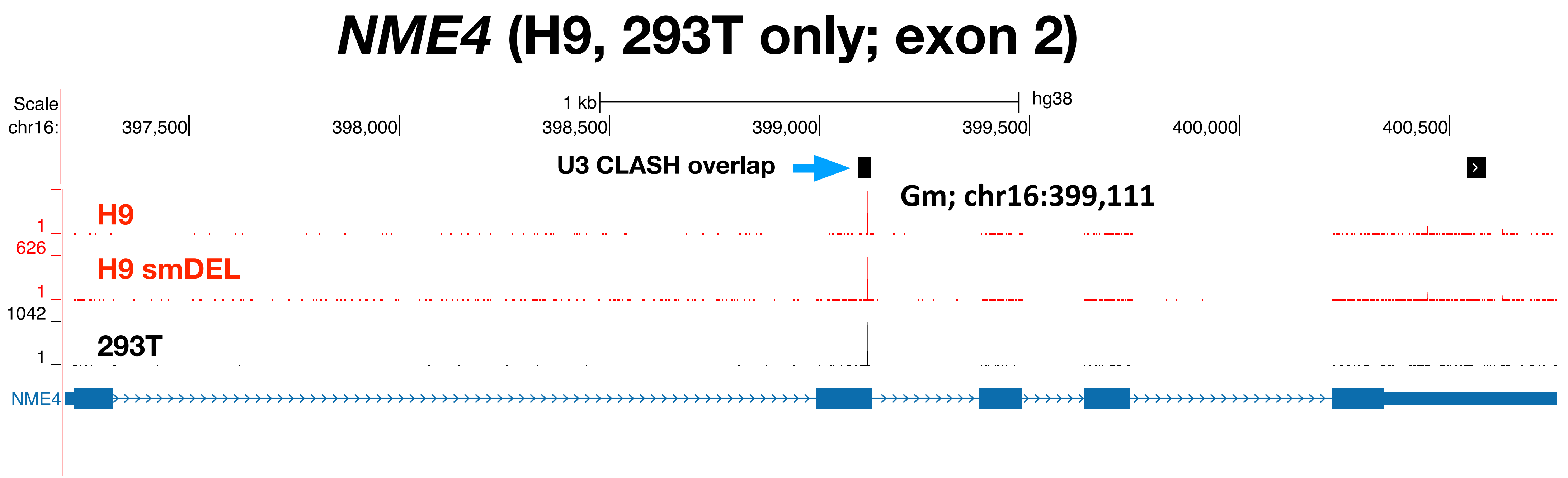
NME4 (H9, 293T only; exon 2)
Scale
chr16:
1 kb
hg38
397,500
398,000
398,500
399,000
399,500
400,000
400,500
U3 CLASH overlap
Gm; chr16:399,111
H9
626
1
H9 smDEL
1042
1
293T
1
NME4

\

N

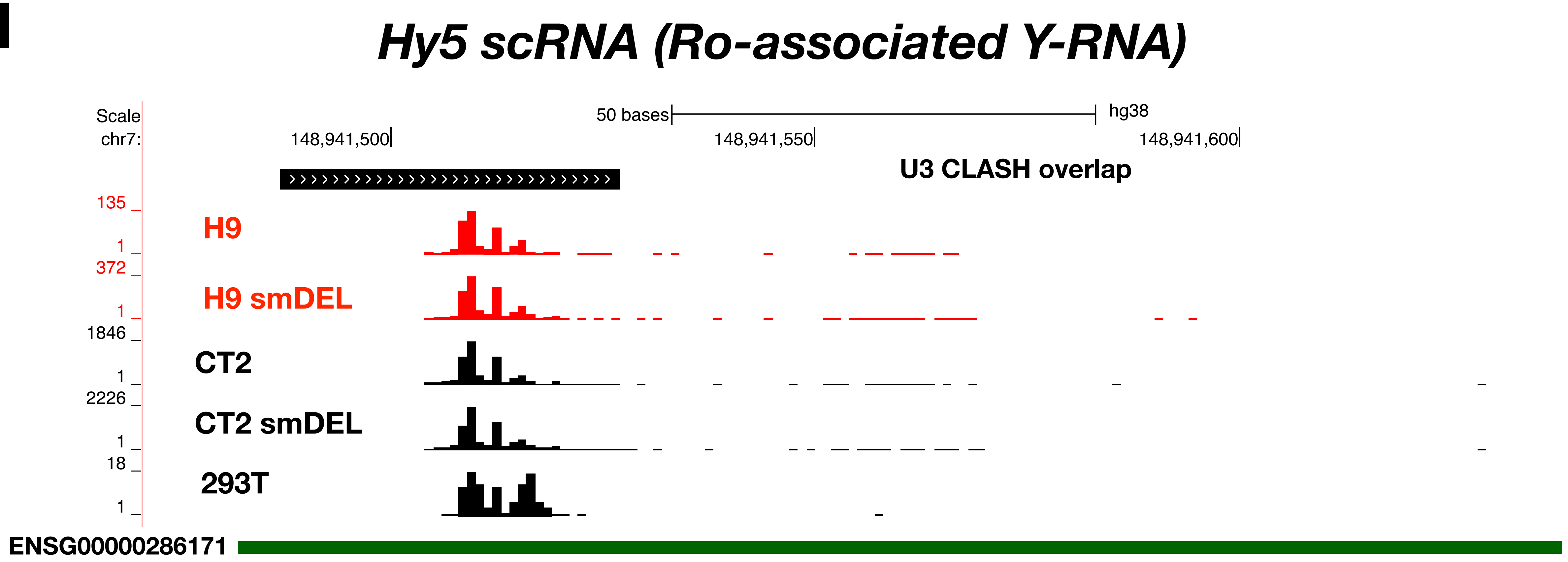
Hy5 scRNA (Ro-associated Y-RNA)
Scale
chr7:
50 bases
hg38
148,941,500
148,941,550
148,941,600
U3 CLASH overlap
135
H9
1
372
H9 smDEL
1
1846
CT2
1
2226
CT2 smDEL
1
18
293T
1
ENSG00000286171
